# PAPerFly: Partial Assembly-based Peak Finder for ab initio binding site reconstruction

**DOI:** 10.1186/s12859-023-05613-5

**Published:** 2023-12-19

**Authors:** Kateřina Faltejsková, Jiří Vondrášek

**Affiliations:** 1 Institute of Organic Chemistry and Biochemistry of the Czech Academy of Sciences, Flemingovo náměstí 542/2, 160 00 Prague, Czech Republic; 2 Computer Science Institute, Faculty of Mathematics and Physics, Charles University, Malostranské náměstí 25, 118 00 Prague, Czech Republic

**Keywords:** ChIP-seq, DNA recognition, Transcription factor, Peak analysis, Algorithm, Graph theory

## Abstract

**Background:**

The specific recognition of a DNA locus by a given transcription factor is a widely studied issue. It is generally agreed that the recognition can be influenced not only by the binding motif but by the larger context of the binding site. In this work, we present a novel heuristic algorithm that can reconstruct the unique binding sites captured in a sequencing experiment without using the reference genome.

**Results:**

We present PAPerFly, the Partial Assembly-based Peak Finder, a tool for the binding site and binding context reconstruction from the sequencing data without any prior knowledge. This tool operates without the need to know the reference genome of the respective organism. We employ algorithmic approaches that are used during genome assembly. The proposed algorithm constructs a de Bruijn graph from the sequencing data. Based on this graph, sequences and their enrichment are reconstructed using a novel heuristic algorithm. The reconstructed sequences are aligned and the peaks in the sequence enrichment are identified. Our approach was tested by processing several ChIP-seq experiments available in the ENCODE database and comparing the results of Paperfly and standard methods.

**Conclusions:**

We show that PAPerFly, an algorithm tailored for experiment analysis without the reference genome, yields better results than an aggregation of ChIP-seq agnostic tools. Our tool is freely available at https://github.com/Caeph/paperfly/ or on Zenodo (https://doi.org/10.5281/zenodo.7116424).

**Supplementary Information:**

The online version contains supplementary material available at 10.1186/s12859-023-05613-5.

## Background

Gene expression regulation is one of the fundamental cell processes. Many mechanisms of this process have been observed; some of them involve specific binding of a transcription factor to a particular short DNA sequence. This process is often studied in vivo by ChIP-seq [1] (chromatin immunoprecipitation followed by high-throughput sequencing). It is also possible to capture the binding site in vitro; a multitude of methods can be used to do so: for example SELEX-seq [2], SMiLE-seq [3] and others [4].

Such an experiment is necessarily followed by a computational analysis of the resulting data. The first step of this analysis consists of read mapping to a reference genome of the studied organism. Subsequently, the analysis follows with “peak calling” – identification of sequences that are significantly enriched in the experimental data [1, 5, 6]. There are a number of computational tools available for these tasks. Mapping of the sequencing reads to the reference genome can be facilitated by Bowtie or BWA mappers [7]. For the peak calling, MACS is a very popular option [8, 9].

This established pipeline assumes that the genome of the studied organism is known. There are a great number of ongoing genome sequencing efforts—genome analysis projects concerning more than 5000 eukaryotic organisms are indicated in the GOLD database [10]. As of February 2022, these analyses were completed and published for more than 550 eukaryotic organisms. At least partial information on 40 000 eukaryotic genomes is also available in the GOLD database. Still, the process of sequencing the complete genome of an organism is an expensive and tedious task. It may not be available for a structurally aberrant genome or for a metagenomic sample.

Recent efforts also show that using a reference genome can introduce some bias into the analysis results, as it appears that genomic diversity cannot be captured by a single reference sequence [11–13]. In the case of human genetic data, great differences can be found in the amount of genetic data for people of different descents, leading to uneven coverage of the human genomic diversity [11].

From the identified peaks, the binding motif can be identified with MEME or other tools [14, 15]. It appears that the area around the binding motif can be meaningful, too. Some indications that a binding motif specificity is influenced by its surroundings have been observed for a multitude of transcription factors [16–18].

Modern approaches to genome assembly translate the sequencing reads to a de Bruijn graph [19]. The genome assembly process is often translated into a search for an Eulerian path in this graph, i.e. a path that traverses all edges in the graph exactly once [20–22].

In this work, a novel algorithm is proposed for binding site reconstruction from the sequences acquired in a ChIP-seq experiment (or any experiment that produces sequences of different enrichment) even without the known reference. Even though there were some attempts at de novo ChIP-seq analysis [23], this is the first specialized algorithm to do so. Our approach allows the user to escape any bias that could have been caused by the reference genome. The ab initio approach to the binding site reconstruction allows the user not to consider the size of the specifically recognized area. Additionally, by omitting the need to use the reference genome we hope to extend the array of organisms for which gene regulation studies can be performed.

## Methods

PAPerFly takes in raw sequencing reads from a ChIP-seq experiment (or similar; our workflow does not use any assumptions about the particularities of the ChIP-seq experiment design) and the size of *k*-mer (the value of *k*) as input and outputs significantly enriched sequences (“peaks”) with their respective significance.

### Algorithm and implementation

**Fig. 1:**
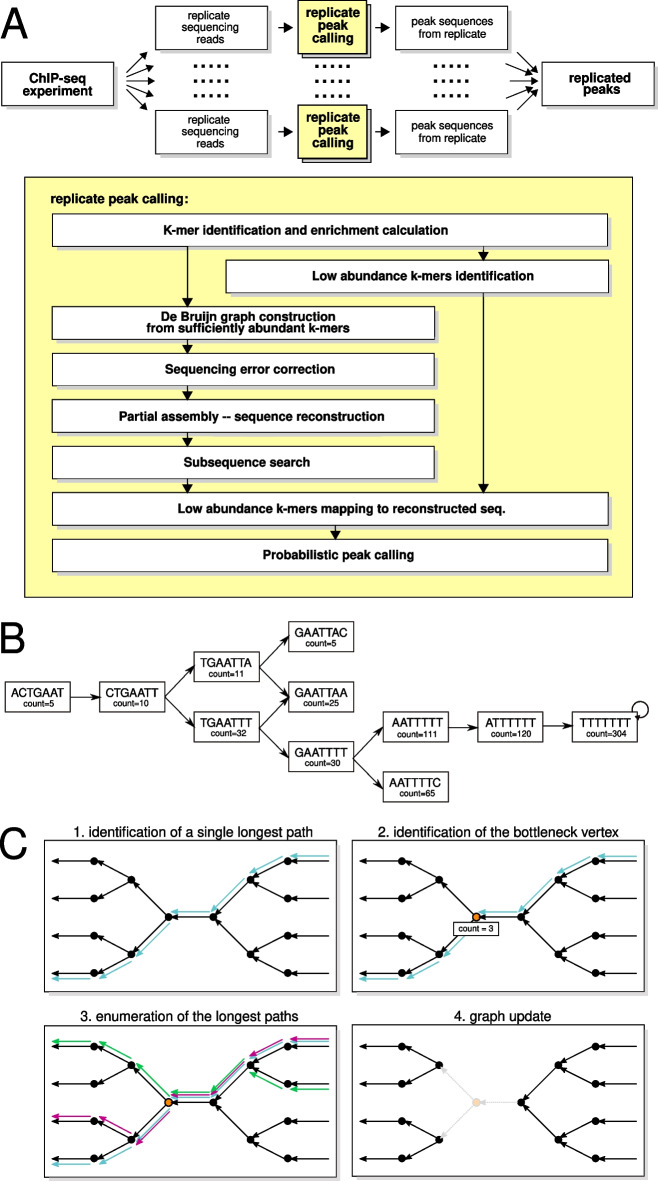
**A** overview of the PAPerFly algorithm. **B**: illustration of a node-centric de Bruijn graph for $k=6$. **C** illustration of bottleneck processing in a single iteration **B** consider two *k*-mers $K_1, K_2$ that are seen in the sequencing data. These *k*-mers constitute nodes of the graph. The nodes $K_1, K_2$ are connected by an oriented edge if and only if the two *k*-mers overlap by $k-1$ (in this case, 6) characters; furthermore, the sequence constructed by contracting the *k*-mers $K_1, K_2$ must occur in the sequencing data. **C** firstly, the longest path in the DAG is identified using a topological sorting of the vertices in the DAG (step 1, path drawn in blue). On it, we identify a bottleneck (step 2, orange vertex). Depending on the bottleneck *k*-mer abundance (in this case, 3), we enumerate as many longest paths as possible while the abundance of each path stays a non-zero integer. Here, we enumerate three paths (step 3, depicted in blue, green and magenta) and assign them abundance of one. Finally, the consumed abundances are subtracted from the *k*-mer counts. The vertices corresponding to zero count *k*-mer are removed. In every step, at least one such *k*-mer exists (step 4, removed edges and vertex are drawn in gray). The process is discussed in more detail in Additional file 1: section S1

The steps of the PAPerFly algorithm are outlined in Fig. 1A. Each replicate in the experiment is processed on its own using the following steps:

- Identification of *k*-mers in the sequencing reads followed by calculation of the number of occurrences of each *k*-mer in the data and correction of the counts with regards to the control experiment
- Identification of the *k*-mers with low abundance and putting them aside
- Construction of a de Bruijn graph (see Fig. 1B) from *k*-mer with sufficient abundance
- Bubble removal to correct sequencing errors and simplify the de Bruijn graph
- Partial assembly of the sequences represented by the weakly connected components of the graph
- Exact string matching of subsequences on longer sequences
- Alignment of the *k*-mers with low abundance to the reconstructed sequences
- Peak identification using a Gaussian hidden Markov model and a Mann-Whitney U test

The outlined steps are discussed below in more detail. An intersection (with some tolerance for errors) of the sequences identified during the replicate peak calling is then given as output.

#### Graph preparation

The first four steps in the proposed workflow can be considered preparation of the de Bruijn graph for the partial assembly. Firstly, the algorithm traverses the sequencing reads with a sliding window of size *k* and identifies the sequences of *k*-mers and their respective numbers of observations. This is done for every replicate separately (a preparatory step for peak calling). The *k*-mer counts of the treatment replicates are then summed. Then, the *k*-mers with a low number of observations are pruned and a de Bruijn graph *G* is constructed from the remaining *k*-mers. The removal of the less frequent *k*-mers aims to eliminate sequencing errors, as well as to strengthen the signal of the studied binding site sequence. We do not map canonical *k*-mers on each other.

For the de Bruijn graph construction, we use BCALM2 [24] (version v2.2.3, git commit 9b7b581). The BCALM2 is able to merge the non-branching paths into a single vertex; this leads to a significant lowering of the number of vertices. As BCALM2 merges canonical *k*-mers, we additionally calculate the abundance of every unique *k*-mer using the jellyfish program [25] (version 2.3.0). The insufficiently abundant *k*-mers are pruned; the vertices merged by BCALM2 can be divided when applying the sufficient abundance criterion. The pruned *k*-mers are later included in the alignment.

The abundance threshold is user-defined; it can be set as an absolute value or it is possible to set the threshold as the *p*-th percentile of the *k*-mer counts (the *k*-mers with a single occurrence are considered sequencing errors and are not included in the *k*-mer counts distribution—we are using the default parameters in BCALM2). During the algorithm testing, the latter option was used, as it allows the estimation of a reasonable threshold without any prior knowledge of the sequencing experiment parameters. By setting this threshold, the user is capable of controlling the program distinguishing capabilities; if the processed ChIP-seq experiment is noisy, setting a high abundance threshold (e. g., 95th, 99th percentile) allows one to mitigate the effect.

Subsequently, the de Bruijn graph *G* is deconstructed into weakly connected components, i.e. connected components of the undirected variant of *G*. Our partial assembly algorithm is based on finding paths in the graph, so each weakly connected component of *G* can be processed separately. This greatly reduces the time and space complexity of the partial assembly and alignment algorithms below. The deconstruction is implemented using the networkx Python library [26] (version 2.5).

The weakly connected components are then subjected to error correction. Short bubbles are removed using a variant of the Tour Bus algorithm proposed for the Velvet genome assembler [21]. As the algorithm can lead to a decrease in connectivity, the deconstruction step is repeated.

#### Partial assembly

During the partial assembly, every weakly connected component *C* of the graph *G* is processed on its own. Firstly, we transform the component *C* to a directed acyclic graph (DAG). Then, a single longest path in the DAG is found (see Fig. 1C, step 1). This is possible in linear time using a topological sorting of vertices.

On it, the *k*-mer with the lowest abundance is identified (a path bottleneck). Using topological sorting, we are able to identify all the longest paths that pass the path bottleneck (Fig. 1C, step 2).

The abundance of the bottleneck is an upper bound on the total abundance of all paths passing through this vertex. If more than one path passes through the bottleneck, the abundance is distributed between them. However, if the total bottleneck abundance *A* is lower than the number of paths passing through it, it is clear that not all of the identified paths were present in the original sequence. In that case, we have to pick at most *A* identified paths and assign them the abundance. Here, we divide the abundance uniformly to as many paths as we can reconstruct while the abundance values remain a positive integer (Fig. 1C, step 3). In doing so, we have “consumed” every usage of the total bottleneck *k*-mer. No other sequence can use the *k*-mer anymore; therefore, we can remove it from the graph (Fig. 1C, step 4).

At this point, we reconstruct the sequences represented by the identified paths. Additionally, for every path we subtract the path abundance from each *k*-mer the path used. We remove those with zero abundance.

The entire partial assembly algorithm (mainly the DAG construction) is discussed in more detail in Additional file 1: section S1. It is implemented in C# and relies heavily on the LINQ library.

#### Subsequence search

Here, the shorter sequences reconstructed during the partial assembly are mapped to longer ones using an exact match string search. If we consider two sequences $s_1, s_2$ such that $s_1$ is a subsequence of $s_2$, the two sequences were acquired during the partial assembly of the same weakly connected component. Therefore, we process the weakly connected components separately during the first step.

In each component, we map the shorter sequences onto the longer sequences using the Aho-Corasick algorithm [27]. As a result of this stage, we acquire a set of the longest reconstructed sequences with information on the abundance for each position. This part is also implemented in C#.

#### Low abundance k-mers mapping

The low abundance *k*-mers are mapped to a best match reconstructed sequence using the NCBI BLAST+ tool [28]. For the BLAST mapping, the program uses words of size 10 and the $10^{-3}$ threshold for the E-value of the hit. If the *k*-mer is sufficiently identical (checked using an identity percentage threshold parameter), the *k*-mer abundance is added to the abundance profile. The user can also filter the *k*-mers to map by thresholding their absolute abundance.

#### Peak calling

From the steps above, we have prepared reconstructed sequences. For every sequence, the abundance of each position is available.

In the first steps of the algorithm, *k*-mer counts were prepared for every replicate in both the treatment and control experiments. For each reconstructed sequence, position abundances are calculated for every replicate. If a *k*-mer is present in multiple reconstructed sequences, the *k*-mer count in replicate is divided between the occurrences in the same proportion as the reconstructed counts. In doing so, an integer matrix *A* of shape $r \times l$ is created for each reconstructed sequence, where *r* denotes the number of replicates (either control or treatment) and *l* denotes the sequence length.

Using a Gaussian hidden Markov model (GHMM), the reconstructed sequences are then broken down into segments corresponding to different GHMM states using the HMMlearn implementation (github.com/hmmlearn/hmmlearn). The resulting state sequence is then smoothed: if a short segment (up to units of base pairs) of state *x* is found between two sufficiently long segments of state *y*, state *x* is in that segment replaced with *y*.

Subsequently, we compare the segment in the array of abundances corresponding to a treatment replicate with the segment in the respective control abundances array segment using a Mann–Whitney U test [29] with Bonferroni correction [30]. We opted for the Mann–Whitney U test because it makes no assumption on the underlying distribution. Although the standard data processing pipeline ChIP-seq peak calling assumes Poisson distribution of the read abundance [8], here, we cannot depend on this assumption due to the nature of the partial assembly process.

If multiple replicates are present in an experiment, the program reports an intersection of the peaks identified in different replicates.

#### Additional remarks

For grammar check of this article, the online tool Grammarly was used (https://app.grammarly.com/, free online version).

## Results

### Method comparison on mouse embryonic stem cells (ESC)

To compare our tool with the previous work of He et al. [23], we replicated their analysis on a ChIP-seq dataset from mouse embryonic stem cells (GSE11431) [31]. First, we simulated an analysis without a known reference genome on the acquired dataset using PAPerFly with *k* set as the read length minus one and with the lowest abundance possible. Then, we mapped the results of our analysis to the mouse reference genome (mm8, to be consistent with the analysis in GSE11431) as well as to the peak sequences identified by a standard peak-calling analysis using the NCBI Blast+ tool. We constrained the query sequences by length – only those longer than 1.5*k* were used for mapping. A sequence identified by PAPerFly is considered mapped if a match was found at least at 95% sequence identity in the target database. A subsequence with such property was sufficient. Based on the ratio between the length of the mapped subsequence and the chiptig length, further filtering was done during the analysis.

Our analysis produces two types of output that both reconstruct the binding site. The first one is the result of the low abundance *k*-mer mapping to the reconstructed sequences (see Fig. 1A, further denoted as PAPerFly chiptigs) and serves as an input to probabilistic peak calling. The term “chiptig” was coined by He et al. [23] and comes from mixing the “chip” abbreviation with the term “contig” used in genome assembly. The second one is the output of probabilistic peak calling (further denoted as PAPerFly peaks). The former constitutes a broader binding site reconstruction, while the latter identifies sequences with high treatment counts.

We started with mapping the PAPerFly chiptigs to the mouse genome. Then, we checked whether the PAPerFly chiptig contained a peak sequence identified by the standard analysis for different mapping cutoffs. A mapping cutoff denotes a minimal portion of the mapped subsequence in the entire PAPerFly chiptig. The results can be found in Table 1. With an 80% mapping cutoff, the average of 62% of PAPerFly chiptigs contains a peak sequence as is found by the standard peak-calling. This exceeds the results of He et al. [23] by almost 10% on average. Furthermore, our results are more consistent. As there is another step to get Papefly peaks from PAPerFly chiptigs, we employed a “link if possible” logic in the PAPerFly chiptigs construction. This could explain some part of the observed error as well as the inconsistency between different mapping cutoffs.

**Table 1:** Results of the PAPerFly chiptigs analysis from mouse ESC ChIP-seq

TF	40%	50%	60%	70%	80%	90%
CTCF	0.503035	0.495266	0.439670	0.350571	0.201262	0.096140
E2f1	0.869511	0.854042	0.837325	0.804391	0.773703	0.725798
Esrrb	0.863810	0.840952	0.805714	0.780476	0.747143	0.699048
Klf4	0.813272	0.811728	0.774691	0.716049	0.621914	0.524691
Nanog	0.676176	0.666924	0.636083	0.576715	0.376253	0.294526
Oct4	0.847377	0.847377	0.798092	0.720191	0.645469	0.588235
STAT3	0.983392	0.978147	0.966783	0.952797	0.925699	0.897727
Smad1	0.250792	0.247625	0.200127	0.132996	0.072198	0.039265
Sox2	0.564948	0.558763	0.523711	0.472165	0.439175	0.391753
Suz12	0.839806	0.839806	0.830097	0.815534	0.786408	0.786408
Tcfcp2I1	0.926601	0.920374	0.913701	0.908808	0.895907	0.876335
Zfx	0.950083	0.934276	0.900166	0.836938	0.763727	0.677205
c-Myc	0.919619	0.919619	0.910082	0.900545	0.877384	0.858311
n-Myc	0.814246	0.756983	0.685754	0.631285	0.575419	0.530726
p300	0.909498	0.894012	0.833448	0.723331	0.631796	0.540950

The columns correspond to different mapping cutoffs—the lowest possible portion of a mapped subsequence of a PAPerFly chiptig to the mouse genome. The fraction is calculated from all sufficiently long PAPerFly chiptigs

As a next step of the analysis, we mapped PAPerFly peaks to the peaks acquired by the standard analysis. If we allow up to 4 mismatches on the edges of the standard analysis peak, more than 75 % of PAPerFly peaks have a match in the standard analysis peak (see Table 2). No length-based filtering was done here.

**Table 2:** Results of the PAPerFly peak analysis from mouse ESC ChIP-seq

TF	Matched PAPerFly peak portion
CTCF	0.650831
E2f1	0.822615
Esrrb	0.692163
Klf4	0.760542
Nanog	0.773144
Oct4	0.790123
STAT3	0.821007
Smad1	0.739212
Sox2	0.779143
Suz12	0.833877
Tcfcp2I1	0.781115
Zfx	0.790626
c-Myc	0.772591
n-Myc	0.719416
p300	0.780683

**Fig. 2:**
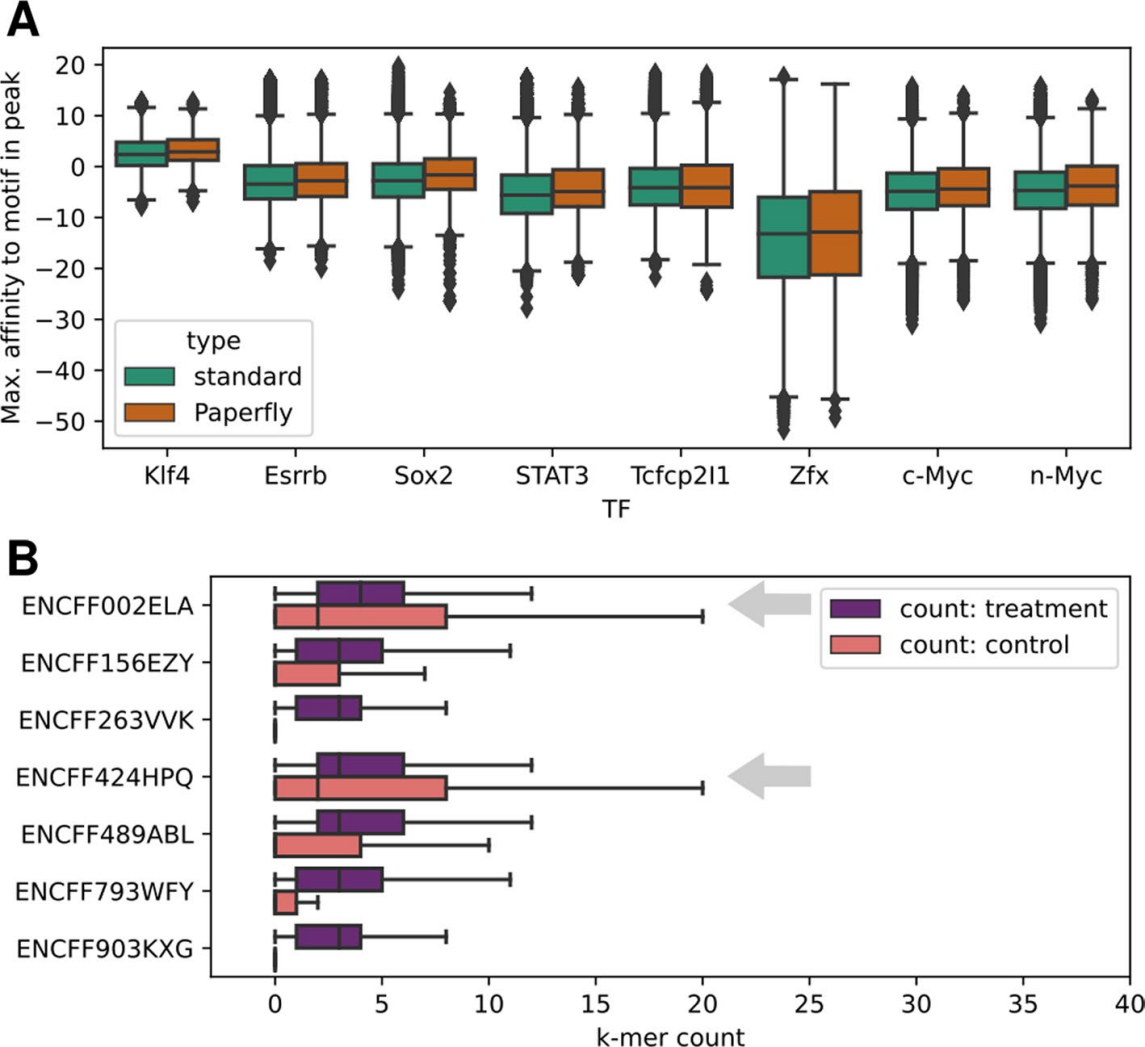
**A**: Boxplot of maximal affinity to the target TF per peak sequence for peak sequences identified by PAPerFly and by the standard analysis for mouse TFs with motifs available in JASPAR. **B**: Count comparison of *k*-mers in control and in treatment *k*-mer sets. The *k*-mers that are present in control but not in treatment are not included. Grey arrows highlight the experiments where PAPerFly achieved very low accuracy. These are the only ones that have higher control *k*-mer count interquartile range than treatment *k*-mer count interquartile range

For the TFs that have a binding site motif without a cofactor in the JASPAR database as of October 2023 (Klf4, Esrrb, Sox2, STAT3, Tcfcp2l1) [32] we calculated the maximal possible affinity for every corresponding PAPerFly peak using Biopython PSSM scoring [33]. We did the same for the peaks identified by the standard analysis. The affinity distribution is similar (see Fig. 2A).

### Human ChIP-seq data, effect of read length

We also tried out our work on human ChIP-seq data. We took the human TF ChIP-seq datasets that were used for testing in the work of He et al. [23]. All of these ChIP-seq experiments used a read length of at most 50bp. To describe the effect of the read length, we took a ChIP-seq experiment with the read length of 100bp for every previously processed TFs. Again, we are using *k* as read length minus one.

As here the peaks identified by the standard peak-calling were much larger than in the mouse dataset and more varied in size, we opted out of the mapping to the human genome and only mapped PAPerFly chiptigs and PAPerFly peaks to the ones from the standard analysis. For the human datasets, we also examine the effect of the size of the chiptig expressed as a function of the read length (see Table 3). We are generally more successful with reconstructing the peak if longer reads are available.

**Table 3:** Results of the PAPerFly chiptigs and peaks analysis from human experiments ChIP-seq

Ident	Read	Chiptigs	Chiptigs	Chiptigs	Chiptigs	Peaks
	Length	$\ge$ *k*	$\ge$ 1.1 *k*	$\ge$ 1.25*k*	$\ge$ 1.5*k*	
ENCFF000QLL	50	0.792559	0.503727	0.292983	0.179104	0.611732
ENCFF000VPU	36	0.441083	0.201490	0.113473	0.058234	0.910155
ENCFF000WDW	36	0.383082	0.191694	0.118622	0.058065	0.931330
ENCFF000XML	36	0.747459	0.528556	0.373114	0.264857	0.836959
ENCFF000YOW	27	0.590401	0.398515	0.386667	0.285714	0.064128
ENCFF000ZBR	36	0.865953	0.723484	0.553480	0.403001	0.807809
ENCFF002EDN	36	0.507555	0.279538	0.193993	0.108176	0.793017
ENCFF002ELA	100	0.090052	0.000000	0.000000	0.000000	0.177778
ENCFF156EZY	100	0.745610	0.649912	0.451807	0.269036	0.612676
ENCFF263VVK	100	0.733179	0.815248	0.668945	0.537217	0.815476
ENCFF424HPQ	100	0.269091	0.336207	0.400000	0.500000	0.157895
ENCFF489ABL	100	0.812528	0.805628	0.645051	0.502525	0.867299
ENCFF793WFY	100	0.889042	0.925232	0.845278	0.779911	0.984674
ENCFF903KXG	100	0.765208	0.914712	0.829531	0.797917	0.989831

In each of the columns, there is the portion of the PAPerFly chiptigs (or peaks) that match a peak sequence as is found by standard peak-calling methods. Only the chiptigs that match a standard peak from at least 75% are counted. For long-read experiments ENCFF002ELA and ENCFF424HPQ and for the short-read experiment ENCFF000YOW, less than 20 PAPerFly chiptigs longer than 1.5*k* were found. For other long-read experiments, lower thousands of chiptigs were reconstructed. Peaks were reconstructed only from chiptigs longer than 1.25*k*

However, reconstructing binding sites from longer reads can be problematic if the control dataset contains highly similar sequences. We encountered this issue with experiments ENCFF002ELA and ENCFF424HPQ where there are similar *k*-mers in the treatment and control database and the (unnormalized) *k*-mer counts in the control dataset exceed those in treatment (see Fig. 2B). Then again, it is easy to identify such a failure even without the validation step: only two PAPerFly chiptigs longer than 1.5*k* were reconstructed for ENCFF002ELA and 16 for ENCFF424HPQ while for others, this number was in the lower thousands (2593 chiptigs on average). A similar failure was observed for the short-read experiment ENCFF000YOW (read length 27), where only 14 chiptigs longer than 1.5*k* was reconstructed. We also attribute this failure to high control *k*-mer counts, however, the differences between this experiment and the high-performing ones are less distinct. ENCFF000YOW is also an experiment with the shortest read length among those used for validation.

Here, we demonstrate using only chiptigs longer than 1.25*k* for PAPerFly peak identification. This option is available in the program. Our validation experiments show that filtering out too short chiptigs increases the program accuracy (from 49.8% (on average) of PAPerFly peaks having a match without filtering to 83.3% with the 1.25*k* filter, not including the failed experiments).

### Processing a multi-replicate experiment

To analyze the performance of the program and the effect of parameters on it, we picked three other human TF ChIP-seq experiments available in the ENCODE database [34] (see Table 4). Each of them was tested for different *k*-mer lengths (21, 25, 31, 35) and *k*-mer abundance cutoffs (99 %, 95 %, 90 %, 80 %).

**Table 4:** ENCODE experiments used for multi-replicate testing and their respective target transcription factor (TF)

Experiment ID	Target TF
ENCSR490LWA	CEBPG
ENCSR065XVO	CHAMP1
ENCSR093FKD	CREB3

To compare the output of PAPerFly with the peak calling analysis that uses the reference genome, we mapped the PAPerFly peaks to corresponding peak sequences deposited in ENCODE using the NCBI BLAST+ tool as described above. To gain more insight into the differences, we measured the mapping success by edit distance from the PAPerFly peak to the ENCODE peak. For edit distance calculation, we used the edlib Python library (version 1.3.9) [35].

**Fig. 3:**
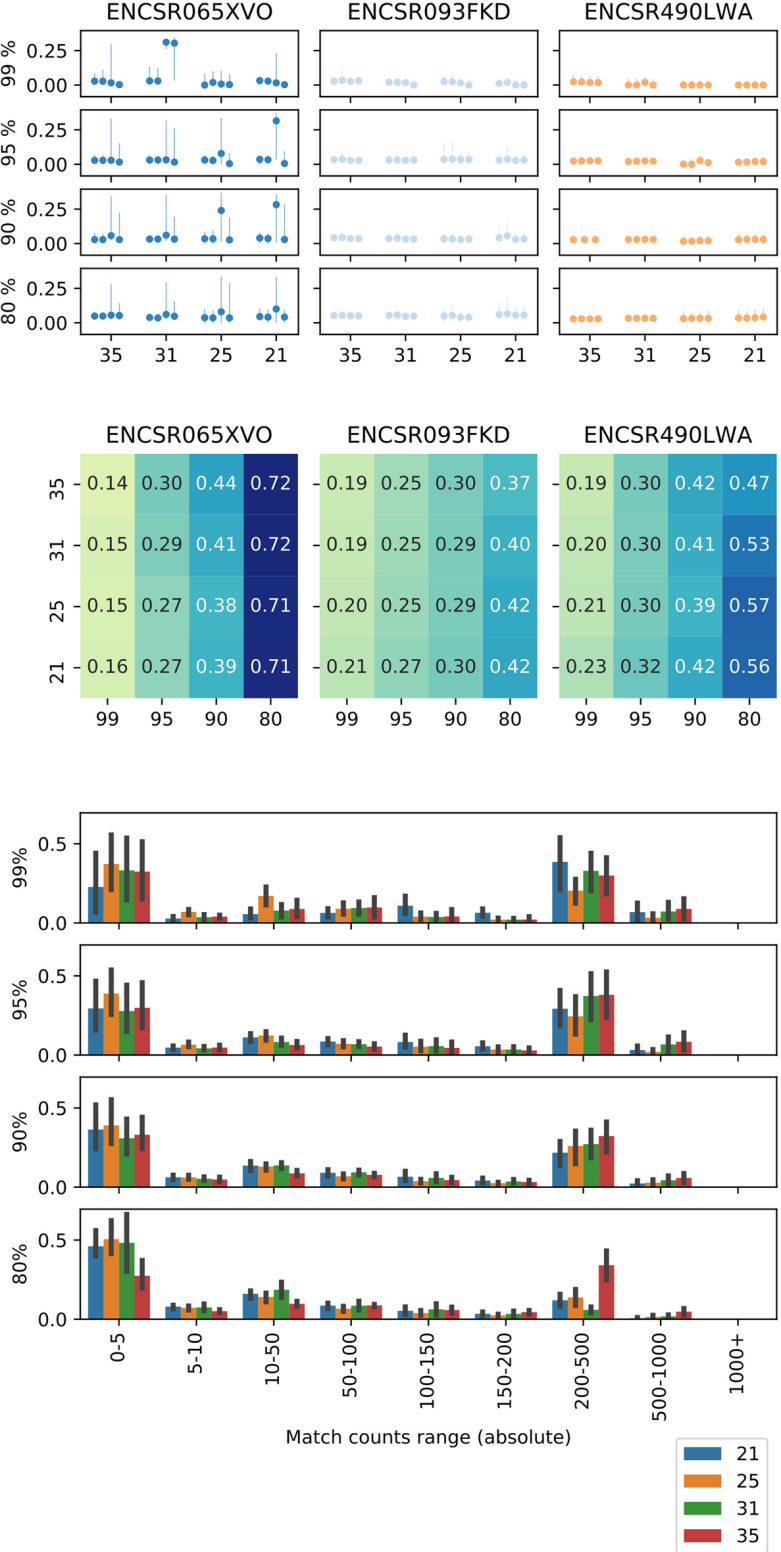
Effect of the *k*-mer size and abundance percentile on the program performance. Top: median edit distance between a replicated PAPerFly peak and matched ENCODE peak (indicated by a point). Vertical lines indicate the interquartile range of the distribution. Middle: heatmaps of portion of ENCODE peak to which a replicated PAPerFly peak is matched corresponding to every tested experiment. Bottom: histograms of the amount of ENCODE peaks mapped to a single PAPerFly peak, estimated from all replicates. Bar height corresponds to the mean fraction of unique peaks in the respective range. Black vertical line crossing the bar denotes the 90 % confidence interval

We have compared the replicated PAPerFly peaks to the peak calling result deposited in the ENCODE database (ENCODE peaks). Results of this comparison can be found in the top plot of Fig. 3. We have observed that *k*-mer size does not affect the mean nor median values of the edit distance between the ENCODE and PAPerFly peak, however, the width of the interquartile range appears to be growing with a lowering of the *k*-mer length, suggesting more errors and incorrectly reconstructed sequences. The edit distance values with both median and mean values around 5 % may be interpreted as high, however, the PAPerFly peaks may be interpreted as consensus sequences of several ENCODE peak sequences (or their section). We have observed that for a sizable portion of the identified peaks, a match can be found in tens or hundreds of ENCODE peaks (see the middle plot in Fig. 3). Approximately half of PAPerFly peaks have only up to five matches in ENCODE peaks. Around a quarter of PAPerFly peaks has more than 150 matches.

## Discussion

Obviously, the time and space complexity of our method depends on the abundance threshold parameter as well as on the *k* parameter. Beyond that, the complexity depends on the sequence variability of the data. The closer the distribution of the sequence *k*-mers is to a uniform distribution, the higher the complexity is. Very noisy ChIP-seq experiments are therefore more time-consuming to process and the results are of low quality (as demonstrated by the processing of ENCFF002ELA and ENCFF424HPQ). Despite that, it is easy to detect such a failure merely by counting the chiptigs longer than 1.5*k*. Other than that, PAPerFly has limitations in processing highly repetitive sequences. In the worst case, the algorithm might misinterpret a highly repetitive sequence as a peak. However, mapping on the reference genome can be similarly limited.

Overall, we were more successful with reconstructing longer patches around the binding site with longer reads. Even if the long enough chiptig is defined in terms of the used *k* (and therefore the experiment read length), a greater portion chiptigs can be mapped to the human genome if longer reads are used. Then again, these experiments are vulnerable if the control *k*-mer counts are similar or higher than those in the treatment *k*-mer set. We intend to continue working on this issue.

Within the testing results, we encountered a non-negligible portion of PAPerFly peaks that can be mapped to a high number of loci that are bound during the ChIP-seq experiment. This observation raises some interesting questions about the specificity of the recognition process. It is possible that the DNA sequence or the DNA topology around the binding motif influences the binding of the transcription factor [16, 17]. The behavior observed in our study may indicate this influence is a part of the recognition done by many (if not all) transcription factors. We intend to continue our focus in this direction.

To conclude, here we present a completely new approach to processing ChIP-seq or similar experiments with our new tool—PAPerFly. Using PAPerFly, we can “assemble” the sequence areas visible in the sequencing experiment and calculate the enrichment of these sequences. All these tasks are performed without any mapping to a reference genome. This tool could extend the array of organisms viable for transcription studies. In addition, it could facilitate transcription studies of an individual without any prior assumptions about the individual’s genome, which may prove useful considering the variability found in human genomes of different background [11], for example, by using the assembled chiptigs as a basis for variant calling.

## Conclusion

We present a new tool to process data from a ChIP-seq or a similar experiment without the need for a reference genome. This tool is based on genome assembly algorithms and on the calculation of *k*-mer enrichment. We processed several ChIP-seq experiments available in the ENCODE database by this tool and compared the results with the peak sequences available in the ENCODE database. As a result, we were able to reconstruct a greater portion of peaks than the tools that use a combination of a genome assembly software (Velvet, SEECER) and a widely used peak calling software (MACS) [23], providing a proof of concept of the newly proposed method. PAPerFly can also process an experiment with multiple replicates. It is interesting to note that we observed that some reconstructed sequences captured in the experiments are present in several ENCODE peaks.

The source codes of PAPerFly are publicly available at https://github.com/Caeph/paperfly or on Zenodo (https://doi.org/10.5281/zenodo.7116424). A docker image is also available on dockerhub (caeph/paperfly).

### Supplementary Information

**Additional file 1:** Detailed description of the partial assembly algorithm.

## Data Availability

The mouse dataset is available in the SRA under the identifier GSE11431. The human datasets analysed during the current study are available in the ENCODE database under the following identifiers: ENCSR490LWA, ENCSR065XVO, ENCSR093FKD, ENCFF000VPU (controlled by ENCFF002ECM), ENCFF002EDN (controlled by ENCFF000PPC), ENCFF000QLL (controlled by ENCFF001HUM), ENCFF000WDW (controlled by ENCFF002ECR), ENCFF000XML (controlled by ENCFF000XOO), ENCFF000YOW (controlled by ENCFF000YOV), ENCFF000ZBR (controlled by ENCFF000YOV), ENCFF263VVK (controlled by ENCFF304HKO), ENCFF489ABL (controlled by ENCFF002DTU), ENCFF156EZY (controlled by ENCFF168MST), ENCFF424HPQ (controlled by ENCFF002DTU), ENCFF903KXG (controlled by ENCFF513OBT), ENCFF002ELA (controlled by ENCFF002EFT), ENCFF793WFY (controlled by ENCFF002EFF).The code is available on Github (https://github.com/Caeph/paperfly) or on Zenodo (https://doi.org/10.5281/zenodo.711642). A docker image is also available on dockerhub (caeph/paperfly).Operating system(s): Unix-based. Programming language: Python, C#, bash. Other requirements: Python 3.8 or higher, jellyfish 2.3.0 (https://github.com/gmarcais/Jellyfish), BCALM 2 version 1.4.1 ( https://github.com/GATB/bcalm). For C# program compilation: Mono JIT compiler version 6.12.0 and NuGet version 6.2.1. License: MS-PL for the C# part of the program, BSD-3-Clause otherwise. Any restrictions to use by non-academics: none.

## Abbreviations

GHMM: Gaussian hidden Markov model
TF: Transcription factor
