# Supplementary material for: PAPerFly: Partial Assembly-based Peak Finder for ab initio binding site reconstruction
Source: BMC Bioinformatics. 2023 Dec 19;24:487. doi: 10.1186/s12859-023-05613-5 (PMC10731698; doi:10.1186/s12859-023-05613-5)
Supplement: Supplementary file 1 — Additional file 1: Detailed description of the partial assembly algorithm. [file 12859_2023_5613_MOESM1_ESM.pdf]

Paperfly: ab initio binding site reconstruction

Kateřina Faltejsková [1,2*], Jiří Vondrášek [1,*]

[1]Institute of Organic Chemistry and Biochemistry of the Czech Academy of Sciences, Flemingovo náměstí 542/2, 160 00 Praha 6, Czech Republic and
[2]Charles University, Faculty of Mathematics and Physics, Computer Science Institute, Malostranské náměstí 25, 118 00 Praha 1, Czech Republic.

1 Partial assembly algorithm

1.1 Partial assembly of a weakly connected component

Consider a weakly connected component W of a de Bruijn graph G. Each vertex in W represents a non-branching sequence of length at least k. Additionally, each vertex is associated with the enrichment of the represented sequence. Enrichment of a sequence is calculated as the minimal k-mer enrichment in the sequence.

Firstly, we construct a condensation graph A of W (explained bellow in detail). The condensation graph A is a directed acyclic graph; therefore, it is possible to find the longest path in this graph in time linear to the number of its vertices.

Consider a longest path P_1 in A. On the path, the enrichment of the least abundant vertex can be considered an upper bound on the enrichment of the path. We denote this vertex a bottleneck vertex. The enrichment of this vertex can serve as an upper bound on the total enrichment of the sequence represented by P_1. If there are more paths $P_2, \ldots, P_x$ of the same lengths as P_1 that share the same bottleneck vertex, the enrichment of the bottleneck vertex is an upper bound on the total enrichment of sequences represented by $P_1, \ldots, P_x$. At the same time, there can be at most 4^{n-1} paths of the same length that pass through the same vertex in W, as every vertex in W can be a source of at most 4 edges and a target of at most 4 edges.

In order to include the maximum variability in partially assembled sequence, the algorithm finds a single longest path in A and identifies its bottleneck. Then for a bottleneck with enrichment e, at most e longest paths that pass through the bottleneck are enumerated. The paths in A are translated to sequences and their total enrichment e is uniformly distributed between them.

In order to find all the sequences represented by W, the algorithm removes the "consumed" k-mers from the graph, the condensation graph A is reconstructed and the path finding is repeated. In every iteration of this algorithm, there is at least one k-mer removed completely (the least abundant k-mer in the bottleneck).

1.2 Condensation construction

Construction of a condensation graph A from W starts with identification of strongly connected components (SCCs) in W (Figure 1, part 2).

A strongly connected component C_H of a directed graph H is defined as a maximal induced subgraph of H such that for every pair of vertices $u, v \in V(C_H)$ there exists an oriented path from u to v and an oriented path from v to u in the C_H.

The SCCs that contain only one vertex are added to A right away. Each SCC with more than one vertex is processed by the following steps. Firstly, Eulerian circuit is found in the SCC if it exists. Otherwise, its approximation is found. Afterwards, vertices incident with edges FROM other SCCs are identified and labeled as in-gates. Similarly, vertices incident with edges

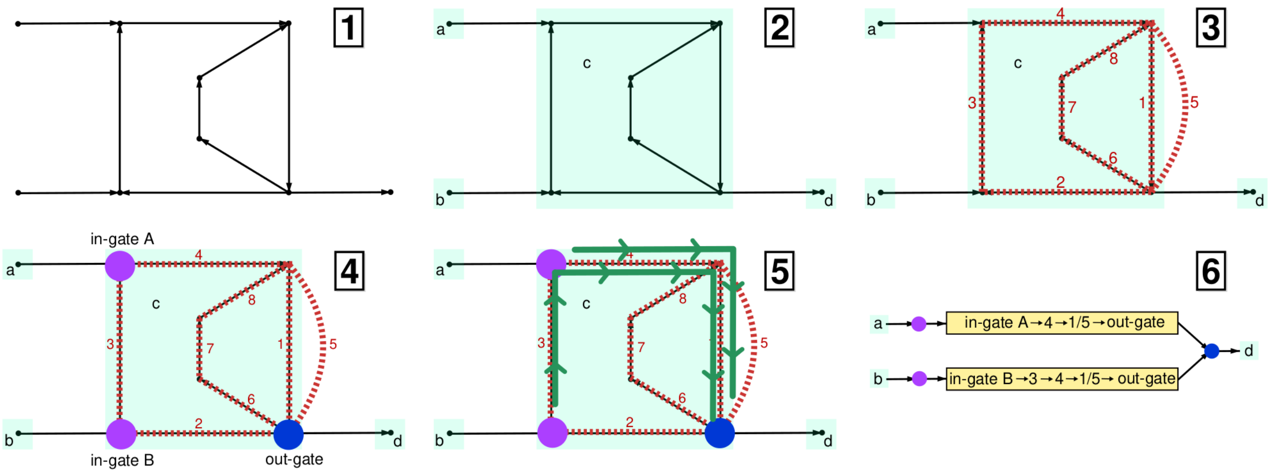

Figure 1: Illustration of condensation construction from a weakly connected component of a de Bruijn graph. **(1)** a weakly connected component of the de Bruijn graph; **(2)** identified strongly components a, b, c, d (highlighted in cyan), only strongly connected component c contains more than one vertex; **(3)** identification of Eulerian circle approximation in c (includes doubling of one edge found by solving an instance of Chinese postman problem; ordering of edges on the cycle is denoted by red numbers); **(4)** identification of in-gates (violet) and out-gates (blue) from and to c; **(5)** finding paths (teal) from an in-gate to an out-gate on the Eulerian circle (for every combination of in-gate and out-gate); **(6)** condensation graph construction, gate vertices and walk-through vertices (yellow) are added to the condensation graph;

TO other SCCs are found and denoted as out-gates. For every pair of in-gate vertex and out-gate vertex, the path from the in-gate to the out-gate is identified as the shortest walk on the Eulerian circuit or its approximation.

All in-gates and out-gates are added to the condensation as vertices that represent no sequence. If a single vertex is both an in-gate and an out-gate, two distinct representation for the vertex are used. A vertex is added for every traversal. This process is illustrated in Figure 1.

If the multivertex SCC has only in-gates, its traversal is for an in-gate a defined as a walk from a to a on the circuit. The traversal is defined similarly if the multivertex SCC has only out-gates. If the multivertex SCC is isolated, the start of the traversal is defined as the least abundant vertex in the component. This is motivated by the intuition that the enrichment of the targeted sequence should follow a distribution similar to normal distribution.

It is possible to find an Eulerian circle in time linear to the number of edges in the graph if the graph contains such a cycle. An oriented graph contains an Eulerian circle if and only if the in-degree of each vertex is equal to its out-degree. Therefore, it is possible to check whether a graph contains an Eulerian path/circle in time linear to the number of nodes in a graph (Fleischner, 1990; Pevzner *et al.*, 2001).

If the graph does not contain an Eulerian circle, its approximation can be defined as the minimal circuit that traverses all the edges in the graph at least once (Edmonds and Johnson, 1973)). A polynomial algorithm is known for this problem if the graph is strongly connected, *i. e.,* if every pair of nodes is connected by a path.

1.3 Complexity

Let N_V be a number of vertices in W and N_K number of k-mers in W. Identification of the strongly connected components in W can performed in $\mathcal{O}(N_V)$ using the Tarjan algorithm (Tarjan, 1972). Tarjan algorithm returns the components topologically sorted.

As for the complexity of construction of SCC traversals, let C_A be a SCC in W and let $V(C_A)$ be the set of its vertices. If the subgraph of C_A is Eulerian, the Eulerian cycle can be found in $\mathcal{O}(|V(C_A)|)$, otherwise, the approximation can be found in $\mathcal{O}(|V(C_A)|^3)$. Additionally, there can be at most $|V(C_A)|^2$ combinations of in-gate and out-gate. For every combination, a walkthrough is found in $\mathcal{O}(|V(C_A)|)$. Therefore, the construction of SCC traversals in C_A can be performed in $\mathcal{O}(|V(C_A)|^3)$. Construction of the condensation graph A from W can be done in $\mathcal{O}(N_V + \sum_{C_A \subseteq A} |V(C_A)|^3)$ where C_A denotes a multivertex SCC in A.

Furthermore, let us consider an iteration of the partial assembly algorithm. The search for the longest path in A can be performed in $\mathcal{O}(V(A))$. Let e_b be the enrichemnt of the bottleneck. Enumeration of feasible paths can be performed in $\mathcal{O}(e_b \cdot V(A))$. As in each iteration at least one k-mer is removed, there is at most N_K iterations.

The update of k-mer counts W performed after filling a bottleneck up can be performed in $\mathcal{O}(N_V)$ as one only needs to update the vertices on one of the identified paths. If the bottleneck was not in a multivertex SCC, the corresponding vertex in the condensation graph can be removed, too. Otherwise, new condensation is constructed.

Let T be the number of vertices in strongly connected component, then complexity of the partial assembly algorithm of W is $\mathcal{O}(N_K \cdot N_V + T \cdot (N_V + \sum_{C_A} |V(C_A)|^3))$.

References

Aho, A. V. and Corasick, M. J. (1975). Efficient string matching: an aid to bibliographic search. *Communications of the ACM*, **18**(6), 333–340.

Edmonds, J. and Johnson, E. L. (1973). Matching, euler tours and the chinese postman. *Mathematical programming*, **5**(1), 88–124.

Fleischner, H. (1990). *Eulerian graphs and related topics*. Elsevier.

Pedregosa, F., Varoquaux, G., Gramfort, A., Michel, V., Thirion, B., Grisel, O., Blondel, M., Prettenhofer, P., Weiss, R., Dubourg, V., Vanderplas, J., Passos, A., Cournapeau, D., Brucher, M., Perrot, M., and Duchesnay, E. (2011). Scikit-learn: Machine learning in Python. *Journal of Machine Learning Research*, **12**, 2825–2830.

Pevzner, P. A., Tang, H., and Waterman, M. S. (2001). An eulerian path approach to dna fragment assembly. *Proceedings of the national academy of sciences*, **98**(17), 9748–9753.

Šošić, M. and Šikić, M. (2017). Edlib: a c/c++ library for fast, exact sequence alignment using edit distance. *Bioinformatics*, **33**(9), 1394–1395.

Tarjan, R. (1972). Depth-first search and linear graph algorithms. *SIAM journal on computing*, **1**(2), 146–160.
